# Dysfunction of cecal microbiota and CutC activity in mice mediating diarrhea with kidney-yang deficiency syndrome

**DOI:** 10.3389/fmicb.2024.1354823

**Published:** 2024-03-04

**Authors:** Mingmin Guo, Leyao Fang, Meili Chen, Junxi Shen, Zhoujin Tan, Wenzhi He

**Affiliations:** ^1^School of Pharmacy, Hunan University of Chinese Medicine, Changsha, China; ^2^School of Traditional Chinese Medicine, Hunan University of Chinese Medicine, Changsha, China; ^3^Changsha Hospital of Traditional Chinese Medicine, Changsha, China; ^4^School of Stomatology, Hunan University of Chinese Medicine, Changsha, China

**Keywords:** adenine, folium sennae, diarrhea with kidney-yang deficiency syndrome, CutC, cecal microbiota, TMAO, inflammatory factors

## Abstract

**Objective:**

Previous studies have indicated that diarrhea with kidney-yang deficiency syndrome leads to a disorder of small intestine contents and mucosal microbiota. However, the relationship of TMA-lyase (CutC) activity and TMAO with diarrhea with kidney-yang deficiency syndrome remains unexplored. Therefore, this study explores the relationship between cecal microbiota and choline TMA-lyase (CutC) activity, as well as the correlation between trimethylamine oxide (TMAO), inflammatory index, and CutC activity.

**Method:**

Twenty SPF-grade male KM mice were randomly divided into the normal group (CN) and the diarrhea model group (CD). Diarrhea mouse models were established by adenine combined with *Folium sennae* administration. CutC activity, TMAO, interleukin-6 (IL-6), and tumor necrosis factor-α (TNF-α) levels were detected, and the cecal content microbiota was sequenced.

**Result:**

After 14 days, diarrhea occurred in the CD group. Compared with the CN group, there was no significant change in the activity of CutC in the small intestine of the CD group, while the activity of CutC in the cecum was significantly increased, and the levels of TMAO, IL-6, and TNF-α showed a significant increase. The Chao1 index, Observed_species index, Shannon index, and Simpson index all exhibited a decreasing trend. The main changes at the bacterial genus level were *Alistipes*, *Enterorhabdus*, *Desulfovibrio*, *Bacteroides*, *Candidatus_Saccharimonas*, and *[Ruminococcus]_torques_group*. The results of LEfSe analysis, random forest analysis and ROC curve analysis revealed *Paludicola*, *Blautia*, *Negativibacillus*, *Paraprevotella*, *Harryflintia*, *Candidatus_Soleaferrea*, *Anaerotruncus*, *Oscillibacter*, *Colidextribacter*, *[Ruminococcus]_torques_group*, and *Bacteroides* as characteristic bacteria in the CD group. Correlation analysis showed a significant negative correlation between cecal CutC activity and *Ligilactobacillus*, and a significant positive correlation with *Negativibacillus* and *Paludicola*. The level of TMAO was significantly positively correlated with CutC activity and IL-6.

**Conclusion:**

Diarrhea with kidney-yang deficiency syndrome significantly affects the physiological status, digestive enzyme activity, CutC activity, TMAO levels, and inflammatory response in mice. Additionally, there are changes in the composition and function of cecal microbiota, indicating an important impact of diarrhea with kidney-yang deficiency syndrome on the host intestinal microbiota balance. The occurrence of diarrhea with kidney-yang deficiency syndrome may be associated with dysbiosis of intestinal microbiota, increased CutC activity, elevated TMAO levels, and heightened inflammatory factor levels.

## 1 Introduction

Diarrhea is defined as more than 3 daily defecations, increased fecal water content, and loose, unformed stool. It is a common global public health issue and the second leading cause of death in children under 5 years old, with 525,000 children dying from this disease every year, which seriously impacts the physical and mental health as well as the quality of life of patients (Schiller et al., 2017; Case and Dickenson, 2018). The earliest discussion of diarrhea in traditional Chinese medicine (TCM) is found in the “Yellow Emperor’s Classic of Internal Medicine.” Due to variations in the etiology, pathogenesis, and clinical manifestations of diarrhea, different TCM patterns emerge, among which the deficiency kidney-yang syndrome was a common syndrome of diarrhea. According to the theory of TCM, the human body is considered an organic whole. When the kidney yang is deficient and fails to warm the spleen yang, the body’s ability to transform dampness properly is compromised. As a result, dampness cannot be adequately processed, leading to diarrhea (Li et al., 2021). The intestinal microbiota plays a crucial role in human health, establishing a dynamic ecological balance between the host and the external environment. Disruption of this balance can lead to various host dysfunctions, such as damaged barrier function, inflammation, and immune function loss, which will induce diseases (Sun et al., 2022; Wang et al., 2022; Xu et al., 2022). Studies have shown that the disorder of intestinal microbiota structure is closely related to the occurrence of diarrhea. Diarrhea is usually accompanied by a disorder of microbiota structure. Compared with non-diarrhea patients, there are significant differences in the composition of intestinal microbiota in diarrhea patients. Non-diarrhea patients show a higher abundance of Bacteroides and a lower abundance of Firmicutes (Sakai et al., 2021; Zhu et al., 2023). Mice with diarrhea caused by a high-fat diet in fatigue state have dysbacteriosis, *Limosilactobacillus* is significantly reduced, and *Anaerotruncus* is significantly increased (Liu et al., 2023). Additionally, previous research by our team revealed that diarrhea with kidney-yang deficiency syndrome changed the structure and function of the intestinal content microbiota in mice, featuring enriched *Lactobacillus intestinalis* and *Bacteroides acidifaciens* (Li et al., 2022a). Thus, it is evident that diarrhea is closely linked to the ecological balance of the intestinal microbiota.

The intestinal microbiota can produce bioactive metabolites, which directly or indirectly affect the physiological function of the host. Disruption of the microbiota structure can reduce the production of beneficial metabolites and increase the accumulation of harmful metabolites. In diarrhea mice induced by a high-fat diet under fatigue, there is a reduction in the production of short-chain fatty acids (SCFAs) (Qiao et al., 2023). Similarly, in diabetic kidney disease patients with imbalanced intestinal microbiota, the production of SCFAs decreases, and the levels of harmful metabolites such as TMAO increase (Tian et al., 2023). Trimethylamine oxide is a metabolite of choline. Choline is converted into trimethylamine (TMA) under the action of CutC produced by intestinal microbiota. TMA is absorbed through the intestinal wall and reaches the liver through the portal vein. In the liver, it is rapidly oxidized to TMAO by flavin containing monooxygenases (FMOs), mainly FMO3 (Wang et al., 2011, 2015). Choline is primarily metabolized anaerobically by CutC, serving as a major precursor for the formation of TMA in the intestinal tract. The generation of TMA predominantly occurs in the cecum, where distinct cecal bacterial populations have been identified, and their proportions have been associated with elevated levels of TMA or TMAO (Schugar and Brown, 2015). In the pathway of TMAO production, there are two important enzymes involved, and the activity of these enzymes can influence TMAO levels. Inhibiting FMO3 activity can lower TMAO levels but leads to TMA accumulation, resulting in the “fish odor syndrome.” Therefore, the activity of CutC produced by the intestinal microbiota is a focal point in researching the reduction of the harmful metabolite TMAO (Messenger et al., 2013; Wang et al., 2015). The contents of the mouse cecum serve as a physiological polymicrobial source of TMA lyase activity, making the cecal microbial community an optimal focus for research (Skye et al., 2018).

TMAO is considered a uremic toxin, and the dose-related relationship between TMAO and the risk of cardiovascular disease was first discovered in 2011 (Wang et al., 2011). Subsequent research on TMAO and human health has expanded, revealing a positive association between TMAO and the development of cardiovascular diseases, metabolic diseases, and chronic kidney disease (Tang et al., 2015; Zhuang et al., 2019; Sánchez-Alcoholado et al., 2020; Tan et al., 2020; Gatarek and Kaluzna-Czaplinska, 2021; Deng et al., 2022). TMAO is involved in the development of cardiovascular diseases and chronic kidney diseases by upregulating the expression of inflammatory factors such as TNF-α and IL-6 (Al-Obaide et al., 2017; Canyelles et al., 2023). The up-regulated expression of the inflammatory factors TNF-α and IL-6 increases the permeability of vascular endothelial cells, leading to intestinal fluid extravasation, which can cause diarrhea (Chen et al., 2022). It can be speculated that TMAO may be involved in the occurrence and development of diarrhea by promoting inflammatory reactions.

Therefore, this study replicated the diarrhea model from our team’s previous research, observed the behavior of mice, detected microbial activity, digestive enzyme activity, and CutC activity, determined the levels of inflammatory factors and TMAO, and analyzed the microbiota of cecal contents. This study analyzed the characteristics of the cecal content microbiota and the changes in CutC activity at different locations. The research explored the relationship between cecal characteristic microbiota and CutC activity, inflammatory factors, and TMAO levels. These findings contribute to a deeper understanding of the pathogenesis of diarrhea and provide important theoretical and experimental foundations for further research and treatment of diarrhea with kidney-yang deficiency syndrome.

## 2 Materials and methods

### 2.1 Animals

To exclude the effect of gender on the intestinal microbiota, only male mice were used in this study (Wu et al., 2022). Twenty male KM mice of SPF grade, weighing 18–22 g, 4 weeks old (Slack Jingda Experimental Animal Co, Ltd., Changsha, China, License No: SCXK (Xiang) 2019-0004) were used. The mice were housed in specific pathogen-free conditions of the Experimental Animal Center of Hunan University of Chinese Medicine (License No: SYXK (Xiang) 2019-0009) under controlled conditions (temperature: 23–25°C, humidity: 50–70%, light/dark cycle: 12 h) with free access to food and water. The feed was provided by the Experimental Animal Center. The experiment was approved by the Animal Ethics and Welfare Committee of Hunan University of Chinese Medicine (permission number: LL2023032901).

### 2.2 Feed

The mouse diet was provided by the Experimental Animal Center of Hunan University of Chinese Medicine and produced by Jiangsu Medisen Biopharmaceutical Co., Ltd. The main nutritional components included moisture, crude protein, crude fiber, crude fat, crude ash, calcium, total phosphorus, lysine, methionine, and cysteine.

### 2.3 Drugs and preparation

Adenine (Changsha Yaer Biology Co., Ltd. Changsha, China, EZ7890C450) and *Folium sennae* (Anhui Pure Traditional Chinese Medicine Co., Ltd. 2,008,232) were used. Adenine suspension was prepared at a dosage of 50 mg/(kg d) with sterile water and prepared just before use. *Folium sennae* decoction was prepared by soaking the *Folium sennae* in an appropriate amount of water, discarding the water after 30 min, adding 5 times the weight of the herbal medicine in water, and boiling it for 30 min. The filtrate was filtered and collected. Next, an appropriate amount of water was added to the residue, boiling was continued for 15 min, and the filtrate was filtered and collected. The two filtrates were combined, and the solution was concentrated to a concentration of 1 g/mL raw herbs and stored at 4°C (Li et al., 2022b).

### 2.4 Animal grouping and intervention

After a 3 days adaptation period, the 20 male KM mice were randomly divided into the normal group (CN) and the diarrhea model group (CD), with 10 mice in each group. The modeling was performed according to the references (Li et al., 2022a, b). Mice in the CD group were gavaged with adenine suspension at a dosage of 50 mg/(kg d), 0.35 mL per time, once a day, for 14 consecutive days. From the 8th day of modeling, the CD group mice were gavaged with *Folium sennae* decoction at a concentration of 1 g/mL of raw herbs, 0.35 mL per time, once a day, for 7 consecutive days. Mice in the CD group were gavaged with an equal volume of sterile water for 14 consecutive days.

### 2.5 Model evaluation criteria

Based on the clinical manifestations of diarrhea with kidney-yang deficiency syndrome, the diagnostic criteria for macroscopic signs in the mouse model include loose stools or undigested grains in the feces, cold extremities, decreased appetite, and body weight, and a lethargic demeanor (Wu et al., 2022).

### 2.6 Behavioral observation of animals

Using CN group mice as a control, the mental status, spontaneous activity, fecal morphology and color, and perianal cleanliness of mice were observed before and after modeling. The average daily diet and water intake were recorded during the modeling period. The body weight, rectal temperature, and fecal water content of the mice were measured and recorded on the 1st, 5th, 9th, and 13th days of modeling. On the 1st, 5th, 9th, and 13th days of modeling, the feces of mice were collected at 9 a.m., weighed, and recorded the wet weight of the feces samples. The samples were dried to constant weight at 110°C, the dry weight was recorded, and the fecal water content was calculated (Qiao et al., 2023).

### 2.7 Sample collection

After the experiment, blood was collected from all mice for enzyme-linked immunosorbent assay (ELISA). After euthanizing the mice, the liver tissue of each mouse was collected in a separate EP tube and stored at −80°C for ELISA. Under sterile conditions, sterile forceps were used to collect intestinal contents for the measurement of microbial activity, digestive enzyme activity, and CutC activity. The entire intestine was then cut open, and washed with physiological saline, and the intestinal mucosa was scraped for the measurement of digestive enzyme activity and CutC activity. For each group, the intestinal contents and mucosa of 5 mice were collected for CutC activity measurement, and an additional 5 mice from each group had their cecal contents collected and labeled in sterile EP tubes, marked, and stored at −80°C for subsequent 16S rRNA high-throughput sequencing (Li et al., 2022b; Qiao et al., 2023).

### 2.8 Measurement of digestive enzyme activity

The collected small intestine contents and mucosa samples from each group were placed in a conical flask containing sterilized distilled water and glass beads, shaken for 30 min to fully dissolve the enzyme protein, centrifuged at 3000 r/min for 15 min, and the supernatant was collected. The enzyme activity in the supernatant was analyzed using a UV–visible spectrophotometer. Lactase activity was determined by the o-Nitrophenyl β-D-galactopyranoside (ONPG) method at a wavelength of 420 nm, protease activity was determined by the Folin phenol method at a wavelength of 660 nm, and cellulase and sucrase activities were determined by the 3,5-dinitrosalicylic acid (DNS) method at a wavelength of 540 nm (Qiao et al., 2022; Zhou et al., 2024).

### 2.9 Determination of microbial activity

The collected small intestinal content samples were prepared into crude enzyme solutions using the same method as in section 2.8. The 2.5 mL Fluorescein diacetate (FDA) stock solution was added to the sterilized phosphate buffer (A solution) at pH 7.6, and the dry sterile test tube was taken. Then 2 mL of solution A and 50 μL of test solution were added to the shaker at 24°C for 90 min, followed by the addition of 2 mL of acetone to terminate the reaction. The blank control was prepared by sequentially adding 50 μL of the test solution and 2 mL of acetone to 2 mL of Solution A. The absorbance was measured at a wavelength of 490 nm, and each sample was measured in triplicate to represent the microbial activity per unit mass of the sample (Yi et al., 2023).

### 2.10 Measurement of CutC activity

The collected small intestine contents, mucosa and cecum content, and mucosa samples were prepared into crude enzyme solutions using the same method as in Section 2.8. Then, the CutC activity was determined by the picric acid-toluene method at a wavelength of 410 nm. This method is based on the catalysis of choline by CutC to produce TMA, which reacts with picric acid to form a yellow quinoline trimethylamine salt. The salt has an absorption peak at a wavelength of 410 nm. Therefore, the absorbance of the solution at 410 nm was measured, and the standard curve method was used for TMA quantitative analysis, to calculate the enzyme activity of CutC (Ferrell et al., 2021; Heng et al., 2021).

### 2.11 ELISA detection

The blood samples collected from each group were placed at room temperature for 30 min, and centrifuged at 3000 r/min for 10 min, and the upper serum was taken and loaded into the EP tube. The liver samples collected from each group were ground using a tissue grinder to obtain the supernatant. The preparation and analysis of samples were carried out according to the instructions of the kit. The IL-6, TNF-α, and TMAO kits were provided by Quanzhou Kenuodi Biotechnology Co., Ltd.

### 2.12 DNA extraction, 16S rRNA gene amplicon sequencing, and sequence analysis

All cecal content samples of the two groups were sent to Shanghai Personal Biotechnology Co., Ltd. for processing. The DNA extraction kit OMEGA Soil DNA Kit (M5635-02) (Omega Bio-Tek, Norcross, GA, United States) was used to extract total microbial genomic DNA from each tube of cecal content samples according to the manufacturer’s instructions, and stored at −20°C for further analysis. The quantity and quality of extracted DNA were determined by a NanoDrop NC2000 spectrophotometer (Thermo Fisher Scientific, Waltham, MA, United States) and agarose gel electrophoresis, respectively. Specific primers targeting the bacterial 16S rRNA V3 + V4 region were used for PCR amplification. The upstream primer 338F (5′-ACTCCTACGGGAGGCAGCA-3′) and the downstream primer 806R (5′-GGACTACHVGGGTWTCTAAT-3′) were used. Quantification of PCR products was performed using the Quant-iT PicoGreen dsDNA Assay Kit, and the libraries were prepared using the Illumina TruSeq Nano DNA LT Library Prep Kit. For the qualified library, NovaSeq 6,000 SP Reagent Kit (500 cycles) was used to perform 2 × 250 bp paired-end sequencing on the Illumina NovaSeq machine. The cecal content microbiota sequencing data have been deposited in the NCBI database: PRJNA1041605.^1^

### 2.13 Bioinformatics analysis

The sequencing data were mainly analyzed using QIIME2 and R software packages (v3.2.0). ASVs were obtained by denoising the original sequencing data and the ASV feature sequences were compared with reference sequences in the SILVA database to obtain the taxonomic information of each ASV. Using QIIME2 software, the sequence numbers of each sample in the ASV abundance matrix were randomly sampled at different depths, and the number of sequences extracted at each depth and the corresponding number of ASVs were used to draw a rarefaction curve (Qiao et al., 2023). The Chao1 index, Observed_species index, Shannon index, and Simpson index were calculated for each sample to compare the richness and evenness of ASVs between different samples. Beta diversity analysis was performed using the Bray–Curtis distance to investigate changes in microbial community structure between samples. Visualization was performed using principal coordinate analysis (PCoA) and non-metric multidimensional scaling (NMDS) methods (Craciun and Balskus, 2012; Li et al., 2022c). Venn diagrams were generated using the “VennDiagram” R package to visualize shared and unique ASVs between samples or groups. QIIME2 software was used to obtain the composition and abundance table of each sample at different taxonomic levels and present the results with a bar chart. The Linear discriminant analysis effect size (LEfSe) method was used to detect differentially abundant taxa across groups using the default parameters (Bray and Curtis, 1957). Random forests analysis was performed on samples from different groups using the default settings of QIIME2. The PICRUSt2 tool was used to predict the functional abundance of samples in the KEGG database, and LEfSe analysis was performed to obtain differentially abundant metabolic pathways between groups (Douglas et al., 2020). Spearman analysis was used to explore the correlation between CutC activity and cecal content microbiota, as well as the correlation between TMAO level and CutC activity and inflammatory factors.

### 2.14 Statistical analysis

SPSS 25.0 software was used for statistical analysis of the experimental data. The results were expressed as the mean ± standard deviation. If the data were normally distributed and the variances were homogeneous, Student’s *t*-test was used. If the data were not normally distributed, the Mann–Whitney U test was used. *p* < 0.05 was considered significant.

## 3 Results

### 3.1 General characteristics of diarrhea with kidney-yang deficiency syndrome mice

**Figure 1:**
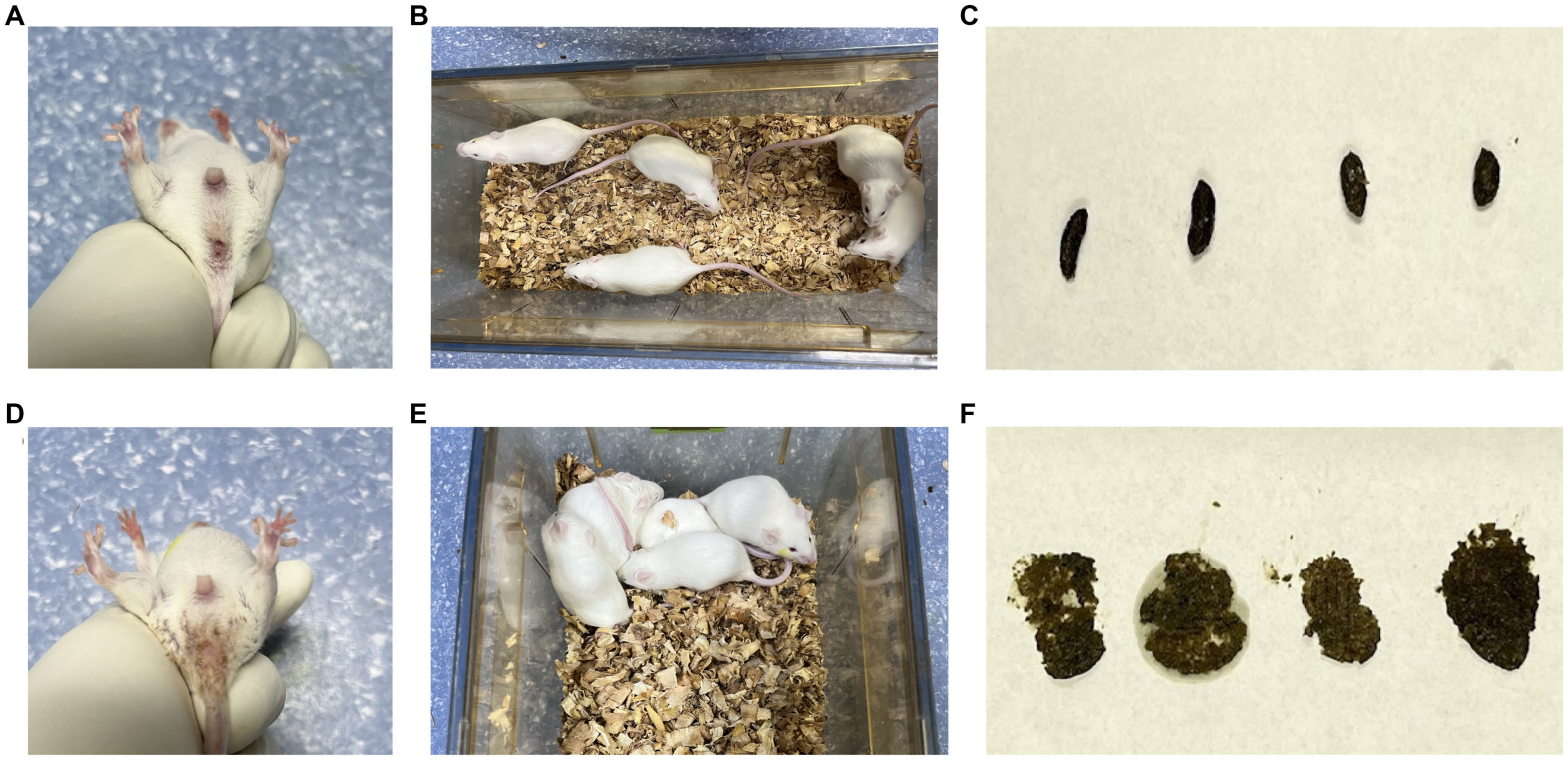
General characteristics of diarrhea with kidney-yang deficiency syndrome mice. **(A)** Perianal condition of mice in CN group; **(B)** mental state and activity of mice in CN group; **(C)** feces of mice in CN group; **(D)** perianal condition of mice in CD group; **(E)** the mental state and activity of mice in CD group; **(F)** feces of mice in CD group.

In the CN group, the mice exhibited normal mental status and spontaneous activity. The bedding was dry, feces were well-formed with moderate consistency, and the perianal area was clean. In the CD group, mice showed signs of poor mental status, reduced spontaneous activity, tendency to gather in clusters, damp bedding, loose and sticky feces that easily adhered to the bedding, and the perianal area was sticky with loose stool (Figure 1). These observations indicate that the modeling procedure altered the behavior of the mice.

**Figure 2:**
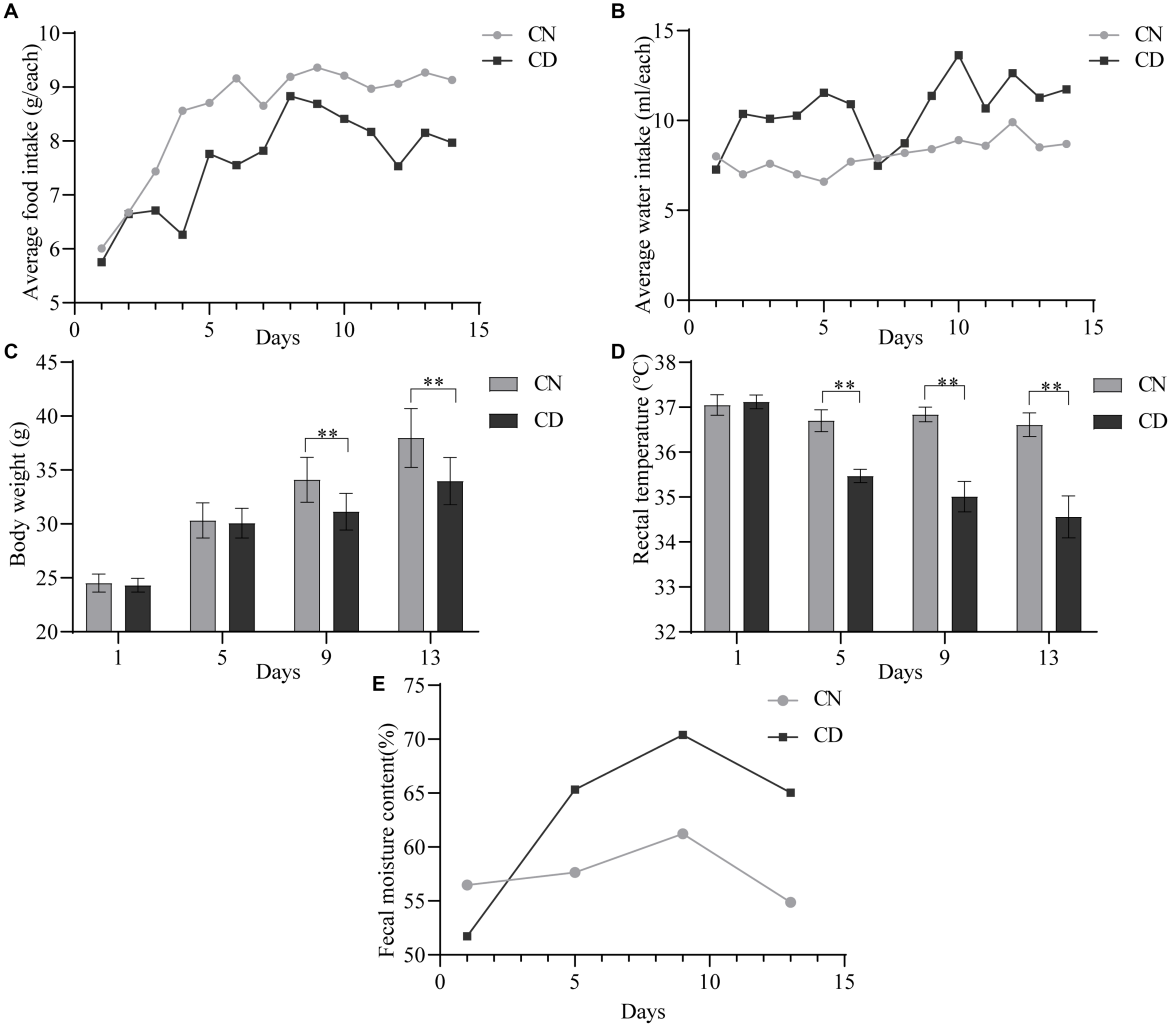
General characteristics of diarrhea with kidney-yang deficiency syndrome mice. **(A)** Average food intake. **(B)** The average water intake. **(C)** Body weight. **(D)** Rectal temperature. **(E)** Fecal moisture content. **p < 0.01. CN, normal group; CD, diarrhea model group.

On the first day of administration, there were no significant differences in the average food intake (Figure 2A) or water intake (Figure 2B) between the CN and CD groups. Starting from the third day of administration, the CD group exhibited a lower daily average food intake than the CN group. From the second day of administration, the CD group showed a higher and more fluctuating daily average water intake, while the CN group’s water intake remained relatively stable. It is suggested that the modeling process suppressed the average food intake in mice and increased their average water intake.

As shown in Figure 2C, there was no significant difference in body weight between the CN group and the CD group on the 1st and 5th days of administration. However, on the 9th and 13th days of administration, the body weight of the CD group was significantly lower than that of the CN group (*p* < 0.01). In Figure 2D, throughout the administration process, the rectal temperature of the CN group showed minimal variation, while the CD group exhibited a larger fluctuation. On the first day of administration, there was no significant difference in rectal temperature between the CN and CD groups. However, on the 5th, 9th, and 13th days of administration, the CD group showed a significant reduction in rectal temperature (*p* < 0.01; *p* < 0.01; *p* < 0.01). This indicates that modeling can inhibit weight gain in mice and cause a decrease in rectal temperature.

As shown in Figure 2E, with an increase in administration time, the fecal water content in the CD group gradually increased and remained higher than that of the CN group from the 5th day onward. This suggests that the modeling process can induce diarrhea in mice.

### 3.2 Intestinal digestive enzyme activity in diarrhea with kidney-yang deficiency syndrome mice

**Figure 3:**
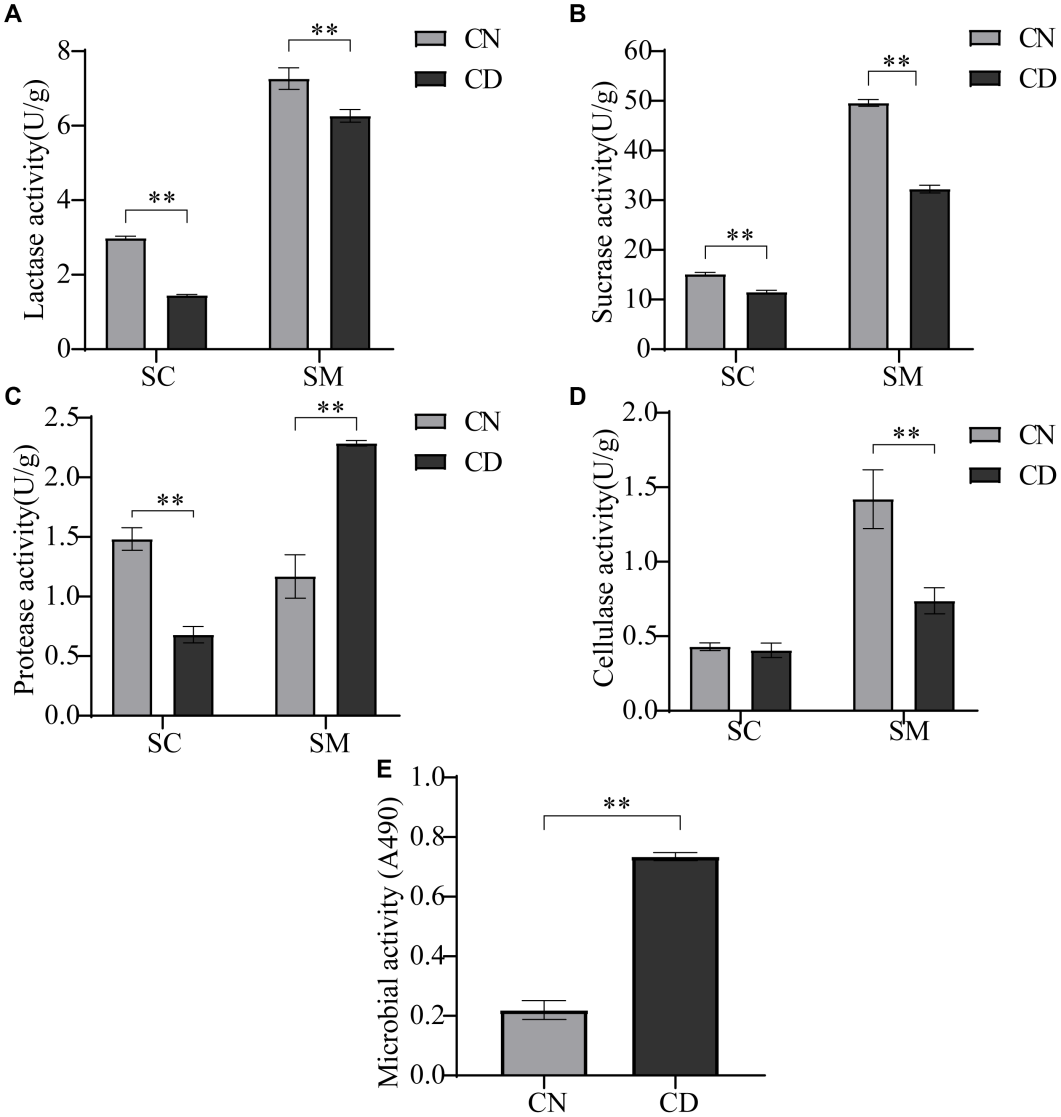
Intestinal digestive enzyme activity and microbial activity in diarrhea with kidney-yang deficiency syndrome mice. **(A)** Lactase activity. **(B)** Sucrase activity. **(C)** Protease activity. **(D)** Cellulase activity. **(E)** Microbial activity. SC, small intestinal contents; SM, small intestinal mucosa. **p < 0.01. CN, normal group; CD, diarrhea model group.

Most nutrients need to be broken down into simpler compounds for absorption by the human body, a process catalyzed by endogenous and exogenous enzymes. As illustrated in Figure 3, the enzymatic activities of lactase, protease, and sucrase in the small intestinal contents of the CD group were significantly lower than those in the CN group (*p* < 0.01), with no difference in cellulase activity. In the small intestinal mucosa of the CD group, lactase, cellulase, and sucrase activities were significantly lower than those in the CN group (*p* < 0.01), whereas protease activity in the small intestinal mucosa of the CD group was significantly higher than that in the CN group (*p* < 0.01). The activity of intestinal digestive enzymes affects the absorption of nutrients by the body. This modeling process reduces the activities of lactase, protease, and sucrase in small intestinal contents, as well as lactase, cellulase, and sucrase in the intestinal mucosa while increasing the activity of protease in small intestinal contents.

### 3.3 Small intestinal content microbial activity in diarrhea with kidney-yang deficiency syndrome mice

The FDA can be catalytically hydrolyzed to generate luminescence by nonspecific enzymes expressed in bacteria and fungi. Microbial activity, to some extent, reflects the metabolic capability of the intestinal microbiota. As shown in Figure 3E, microbial activity in small intestinal contents under conditions of absorbance at 490 nm was significantly higher in the CD group than in the CN group (*p* < 0.01). This suggests that modeling may have promoted the expression of certain microbes or their functional genes.

### 3.4 Intestinal CutC activity in diarrhea with kidney-yang deficiency syndrome mice

CutC plays a crucial role in the synthesis of TMA, a precursor compound for TMAO (Segata et al., 2011). As indicated in Table 1, after diarrhea was induced by adenine combined with *Folium sennae*, the CutC activity in the small intestinal contents of the CN group was slightly higher than that in the CD group, while the enzymatic activity in the small intestinal mucosa of the CN group was slightly lower than that in the CD group. However, the CutC activity in the cecum contents and mucosa of the CD group was significantly higher than those in the CN group (*p* < 0.05). This suggests that the modeling process has a minimal impact on CutC activity in small intestinal contents and mucosa but can enhance CutC activity in cecum contents and mucosa.

**Table 1:** Intestinal CutC activity in diarrhea with kidney-yang deficiency syndrome mice (−*x* ± s, *N* = 3).

Group	CutC activity (U/g)			
	SC	SM	CC	CM
CN	0.90 ± 0.05	0.79 ± 0.17	0.28 ± 0.12	1.01 ± 0.26
CD	0.86 ± 0.12	0.89 ± 0.16	0.97 ± 0.22^a^	1.82 ± 0.18^a^

SC, small intestinal contents; SM, small intestinal mucosa; CC, cecum contents; CM, cecum mucosa. CN, normal group; CD, diarrhea model group.

a Compared with the CN group, *p* < 0.05.

### 3.5 Serum levels of IL-6, TNF-α, TMAO, and hepatic TMAO in diarrhea with kidney-yang deficiency syndrome mice

**Figure 4:**
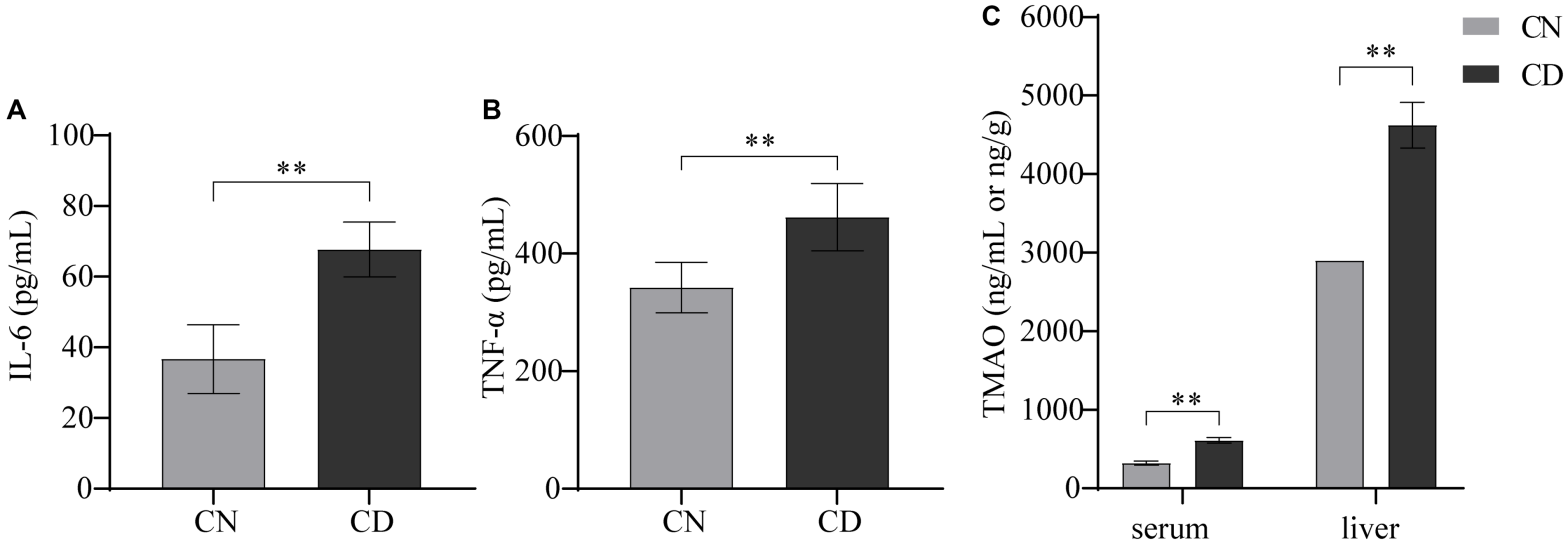
Levels of IL-6, TNF-α, and TMAO in diarrhea with kidney-yang deficiency syndrome mice. **(A)** IL-6 levels. **(B)** TNF-α levels. **(C)** TMAO levels. **p < 0.01. CN, normal group; CD, diarrhea model group.

In Figure 4, compared with the CN group, the CD group exhibited a significant increase in TNF-α (*p* < 0.01) and a highly significant increase in IL-6 (*p* < 0.01) in serum. The levels of TMAO in both serum and liver tissue were significantly elevated in the CD group compared to the CN group (*p* < 0.01), with the TMAO content in the liver being higher than that in serum for both groups. This suggests that the modeling process increased inflammation and TMAO production in mice.

### 3.6 Richness and diversity of cecal contents microbiota in diarrhea with kidney-yang deficiency syndrome mice

**Figure 5:**
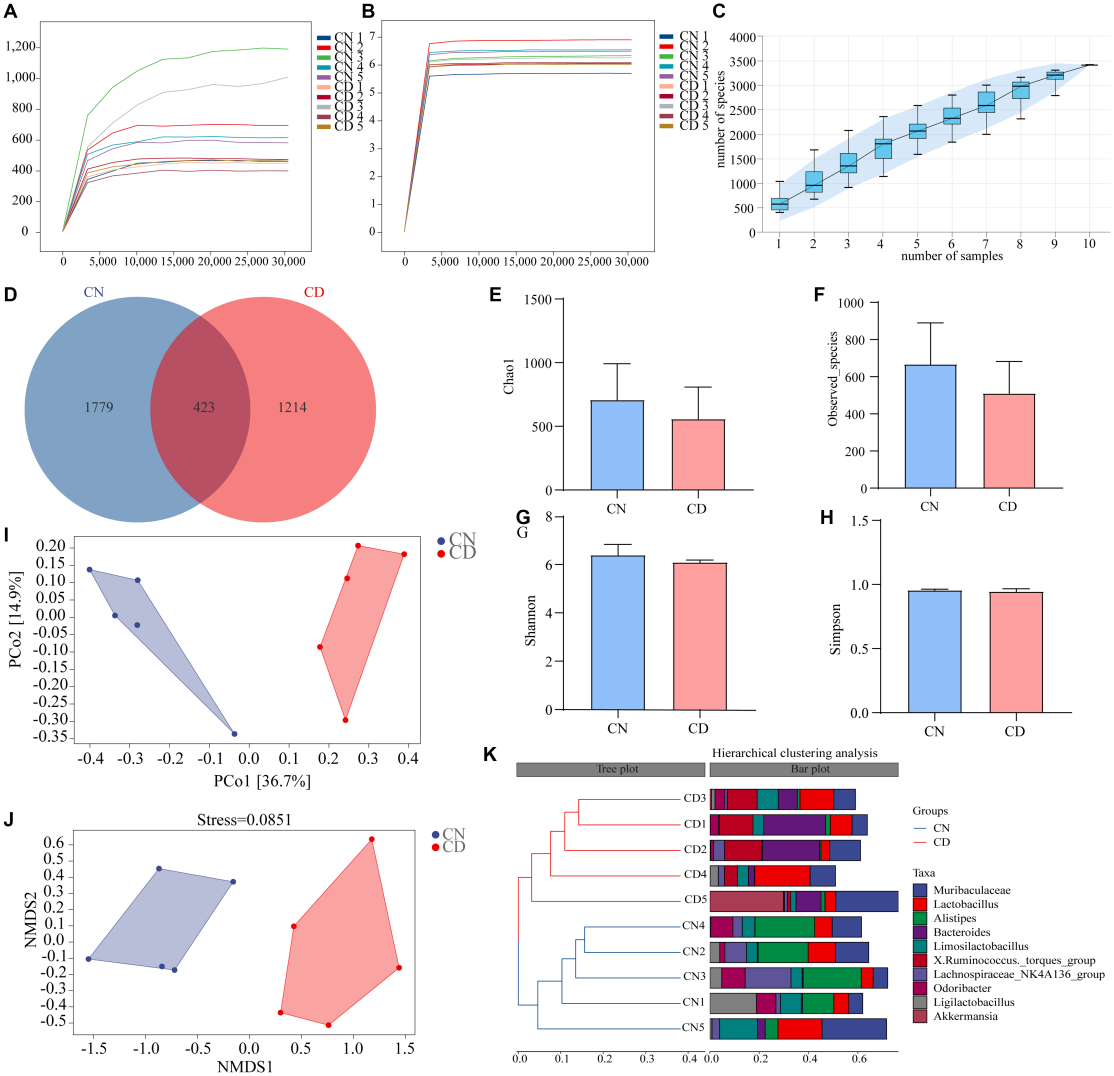
Analysis of richness and diversity of cecal contents microbiota in mice. **(A)** Chao1 dilution curve. **(B)** Shannon dilution curve. **(C)** Species accumulation curve. **(D)** ASV quantity. **(E)** Chao1 index. **(F)** Observed_Species index. **(G)** Shannon index. **(H)** Simpson index. **(I)** PCoA analysis. **(J)** NMDS analysis. **(K)** Cluster analysis. CN, normal group; CD, diarrhea model group.

The dilution curve is primarily used to assess whether the sequencing depth is reasonable, indirectly reflecting the richness of species in the sample. As the sequencing depth of the samples increased, the dilution curve no longer significantly rose and reached a plateau, indicating that the sequencing depth for both groups essentially covered all the species in the samples (Figures 5A,B). Species accumulation curves are utilized to evaluate the adequacy of the sample size. As depicted in Figure 5C, with an increase in sample size, the total number of ASVs no longer significantly rises, and the curve tends to plateau. This indicates that the sample size in this study is sufficient to adequately represent the species composition of the community.

According to Figure 5D, the CN group had 2,202 ASVs, while the CD group had 1,637 ASVs. This result suggests that diarrhea with kidney-yang deficiency syndrome may lead to a reduction in the number of ASVs. Alpha diversity reflects the richness and diversity of species within individual samples. Species richness was evaluated using the Chao1 and Observed_species indices, while species diversity was assessed using the Shannon and Simpson indices. The results indicate a decreasing trend in Chao1 (Figure 5E), Observed_species (Figure 5F), Shannon (Figure 5G), and Simpson (Figure 5H) indices in the cecal contents of mice in the CD group compared to the CN group. This suggests that the alpha diversity of the microbial community within the samples changed during the modeling process, showing a decreasing trend in richness and diversity.

The beta diversity focuses on differences between samples. Principal coordinates analysis projects the distance matrix of samples into a low-dimensional space, preserving the original distance relationships. In Figure 5I, the contribution rate of the *x*-axis PCo1 was 36.7%, and the contribution rate of the *y*-axis PCo2 was 14.9%. The CN group and CD group were non-overlapping and distant, indicating a significant change in the cecal microbial community structure after modeling. Additionally, the results of NMDS are consistent with PCoA, and the stress value of NMDS (0.0851) is considerably less than 0.2, signifying the reliability of the NMDS analysis results. Cluster analysis (Figure 5K) revealed that samples within both the CN and CD groups clustered well together, with minor differences within each group but substantial differences between groups. The results of alpha and beta diversity analyses indicate changes in the richness, diversity, and overall structure of the cecal microbial community in mice after modeling.

### 3.7 Species composition and abundance of the cecal microbial community in diarrhea with kidney-yang deficiency syndrome mice

**Figure 6:**
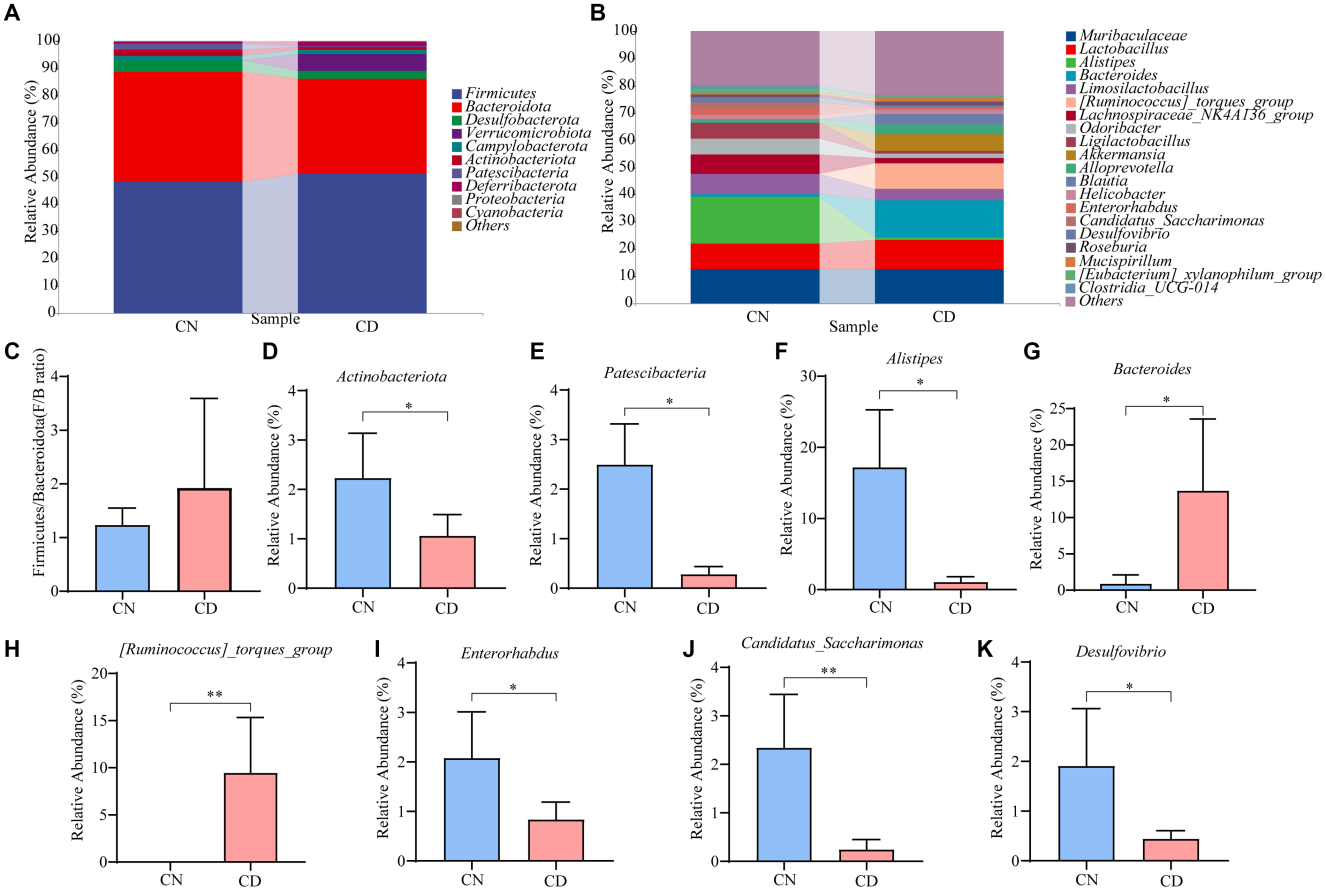
Cecal microbiota species composition and abundance in mice. **(A)** Phylum-level cecal microbiota composition. **(B)** Genus-level cecal microbiota composition. **(C)** F/B ratio. **(D,E)** Dominant phyla in the cecal contents of the CN and CD groups. **(F–K)** Dominant genera in the cecal contents of the CN and CD groups. CN, normal group; CD, diarrhea model group. *p <0.05;**p < 0.01.

Figure 6A displays the relative abundance of cecal microbiota at the phylum level. Firmicutes and Bacteroidetes were the predominant phyla in both the CN and CD groups. Compared to the CN group, the CD group showed a slightly increased Firmicutes/Bacteroidetes (F/B) ratio (*p* > 0.05), with a significant increase in the relative abundance of *Verrucomicrobiota* (*p* < 0.01). In Figure 6B, the top 20 genera at the cecal microbiota level are presented. *Muribaculaceae*, *Lactobacillus*, *Alistipes*, *Bacteroides*, *Limosilactobacillus*, and *[Ruminococcus]_torques_group* are major genera in the cecal microbial community of mice. Compared to the CN group, the CD group exhibited a significant reduction (*p* < 0.05) in *Alistipes*, *Enterorhabdus*, and *Desulfovibrio*, a highly significant decrease (*p* < 0.01) in *Candidatus_Saccharimonas*, a significant increase (*p* < 0.05) in *Bacteroides*, and a highly significant increase (*p* < 0.01) in *[Ruminococcus]_torques_group*. This suggests that the major changes in the cecal microbial community of mice after modeling involve *Actinobacteriota*, *Patescibacteria*, *Alistipes*, *Enterorhabdus*, *Desulfovibrio*, *Bacteroides*, *Candidatus_Saccharimonas*, and *[Ruminococcus]_torques_group*.

### 3.8 Distinctive microbial community in the cecal contents of diarrhea with kidney-yang deficiency syndrome mice

**Figure 7:**
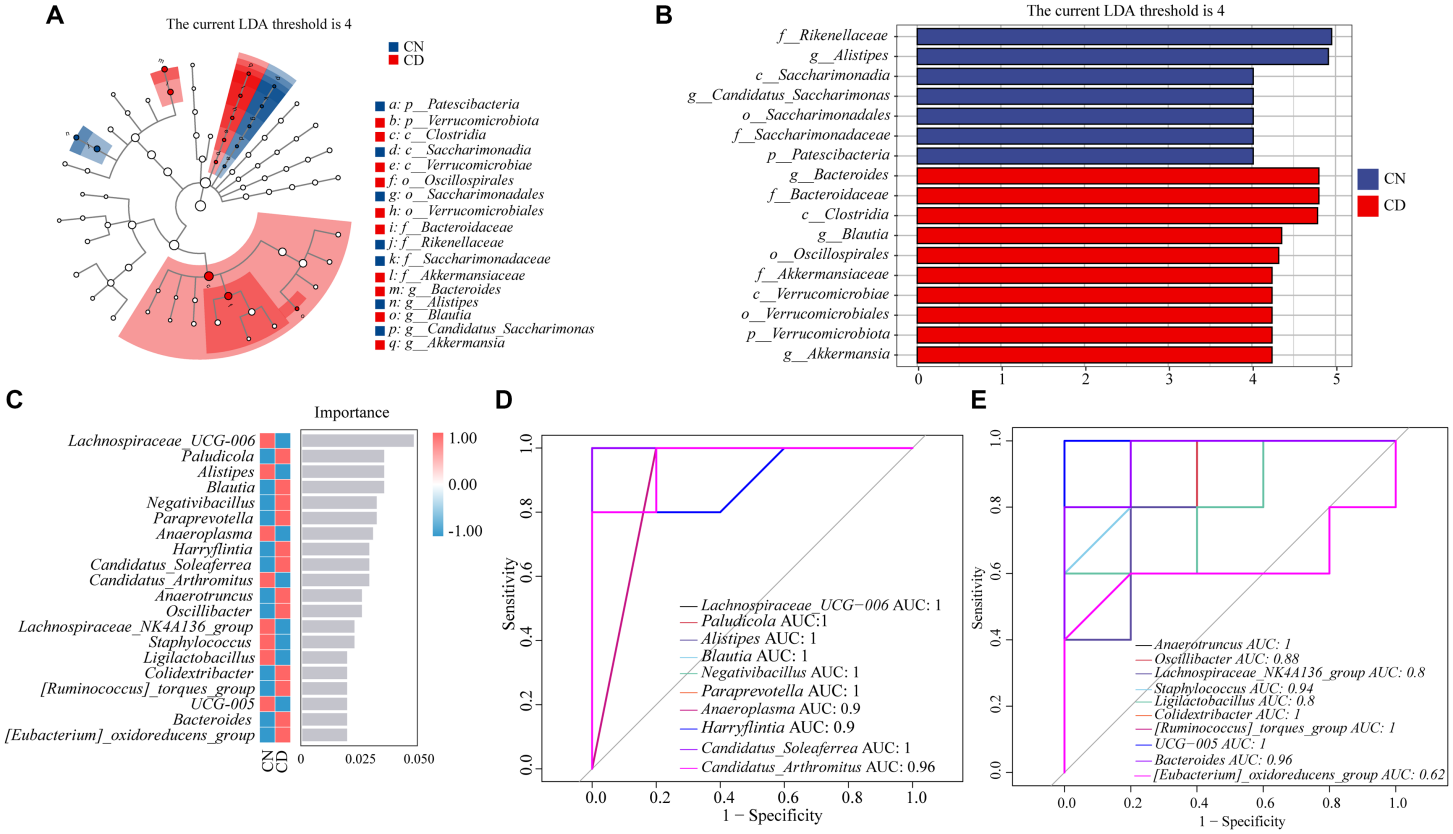
Analysis of core feature microbial groups in the cecal contents of mice. **(A)** Cladogram diagram. **(B)** LDA diagram. **(C)** Random forest diagram at genus level. **(D,E)** ROC curve of genera. CN, normal group; CD, diarrhea model group.

As shown in Figures 7A,B, LEfSe analysis, with a threshold set at >4, was employed to identify genera with significant differences in abundance between groups. Linear discriminant analysis (LDA) was applied to discriminate genera showing significant enrichment between the CN and CD groups. Notably, *Alistipes* and *Candidatus_Saccharimonas* were significantly enriched in the CN group, while *Bacteroides*, *Blautia*, and *Akkermansia* were notably enriched in the CD group.

To identify key species that differentiate between the CN and CD groups, a random forest diagnostic model was constructed using 20 genera, revealing nonlinear relationships among variables (Figure 7C). Subsequently, ROC curve analysis was conducted with an area under the curve (AUC) >0.8 as the criterion (Figure 7D). The characteristic genera in the CD group, including *Paludicola* (AUC = 1), *Blautia* (AUC = 1), *Negativibacillus* (AUC = 1), *Paraprevotella* (AUC = 1), *Harryflintia* (AUC = 0.9), *Candidatus_Soleaferrea* (AUC = 1), *Anaerotruncus* (AUC = 0.9), *Oscillibacter* (AUC = 0.88), *Colidextribacter* (AUC = 1), *[Ruminococcus]_torques_group* (AUC = 1), and *Bacteroides* (AUC = 0.96), exhibited large AUC values, suggesting their distinctive enrichment in diarrhea with kidney-yang deficiency syndrome. Combining the results of LEfSe analysis, random forest analysis, and ROC curve analysis, it can be inferred that *Paludicola*, *Blautia*, *Negativibacillus*, *Paraprevotella*, *Harryflintia*, *Candidatus_Soleaferrea*, *Anaerotruncus*, *Oscillibacter*, *Colidextribacter*, *[Ruminococcus]_torques_group*, and *Bacteroides* constitute characteristic microbial groups in this diarrhea model.

### 3.9 Functional profile of the cecal microbiota in diarrhea with kidney-yang deficiency syndrome mice

**Figure 8:**
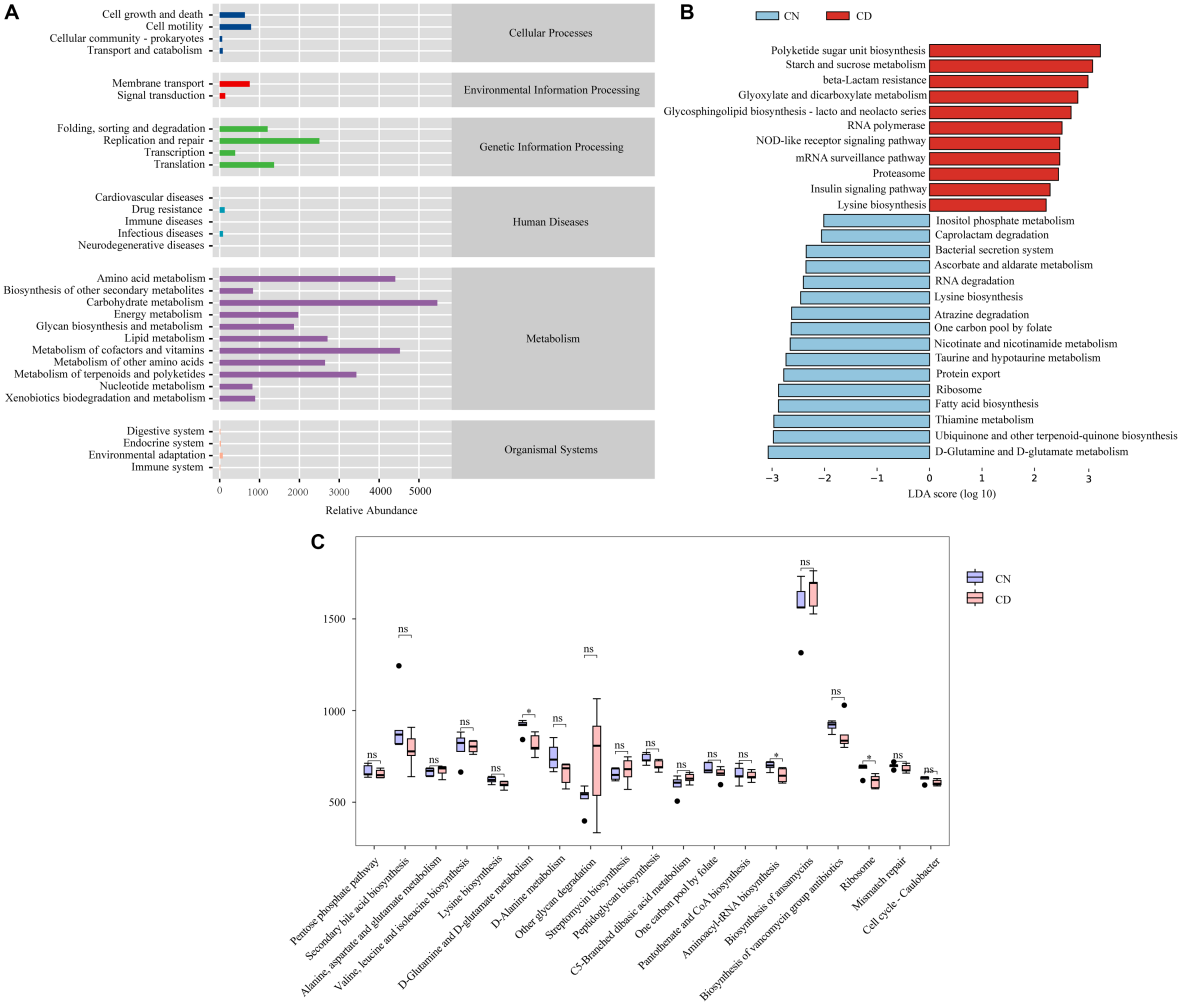
Metabolic function prediction of cecal microbiota based on the KEGG database. **(A)** Predicted abundance of KEGG functions. **(B)** LEfSe analysis results. LDA > 2, *p* < 0.05. **(C)** Inter-group comparison of metabolic functions. *p < 0.05. CN, normal group; CD, diarrhea model group.

To determine the effect of diarrhea with kidney-yang deficiency syndrome on the metabolic function of the bacterial microbiota in the cecal contents of mice, PICRUSt2 analysis based on the KEGG database was employed. Figure 8A shows 6 main functional types composed of 29 functional pathways, among which the metabolic type has the greatest abundance. LEfSe analysis (set threshold > 2) was conducted on all functional pathways to identify those significantly enriched in either the CN or CD group (Figure 8B). Polyketide sugar unit biosynthesis, Starch and sucrose metabolism, beta-Lactam resistance, Glyoxylate and dicarboxylate metabolism, RNA polymerase, Glycosphingolipid biosynthesis-lacto and neolacto series, mRNA surveillance pathway, NOD-like receptor signaling pathway, Proteasome, Insulin signaling pathway, Lysine degradation pathways were significantly enriched in the CD group. In Figure 8C, among the 19 pathways with an abundance greater than the median value of 405.0519 in the third-level metabolic pathways, D-Glutamine and D-glutamate metabolism, Aminoacyl-tRNA biosynthesis, and Ribosome pathways were significantly decreased in the CD group compared to the CN group (*p* < 0.05).

### 3.10 Correlation analysis of intestinal CutC activity and cecal content microbiota, TMAO, and inflammatory factors

**Figure 9:**
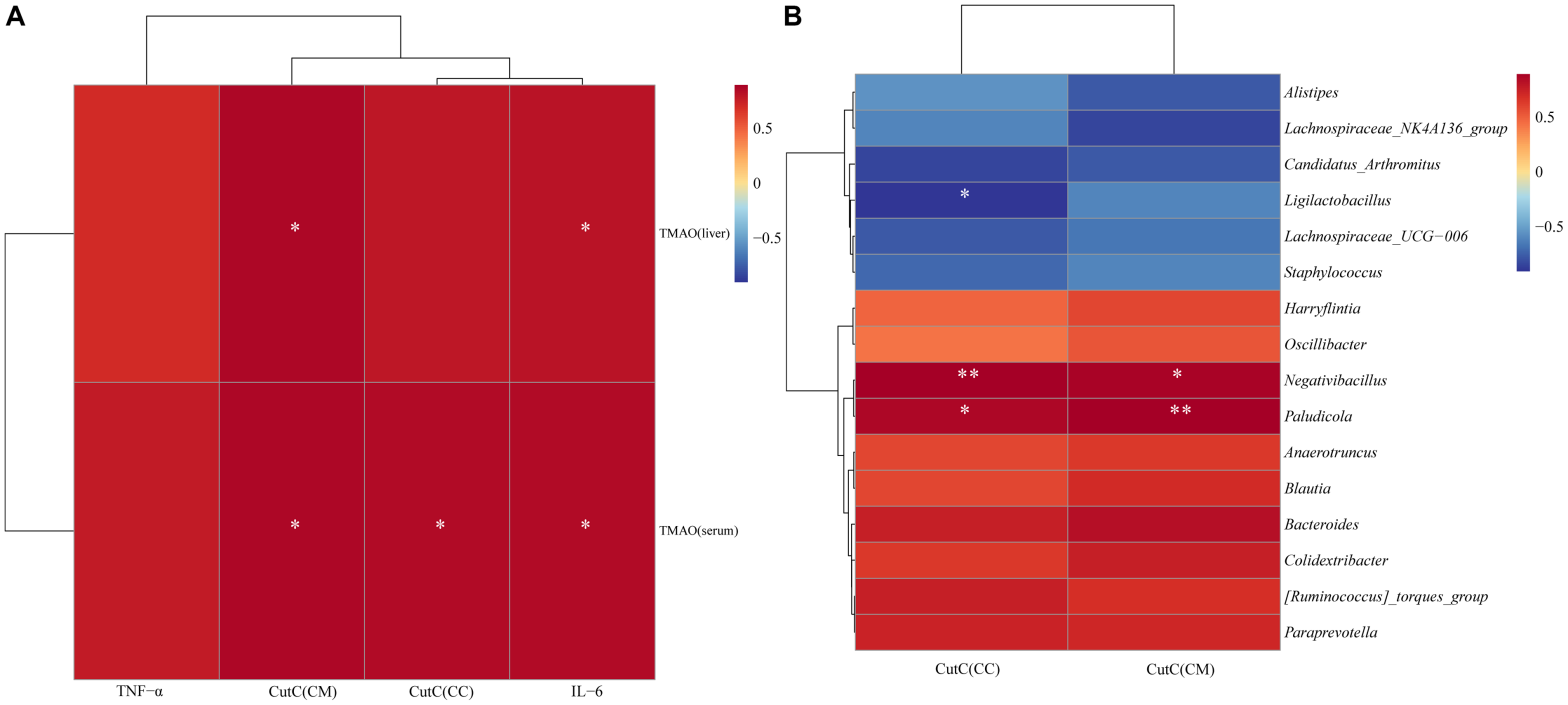
Spearman correlation analysis heatmap: red indicates a positive correlation, and blue indicates a negative correlation. The closer the color is to red, the stronger the positive correlation between two parameters; the closer the color is to blue, the stronger the negative correlation between two parameters. **(A)** Heatmap depicting the correlation of CutC activity, TNF-α, IL-6 in cecal contents (CC) and mucosa (CM) with serum and liver TMAO levels. **(B)** Heatmap illustrating the correlation between CutC activity in cecal contents and mucosa with the feature microbial genera in cecal contents.

To explore the relationship between intestinal CutC activity and cecal content microbiota, TMAO, and inflammatory factors in mice with diarrhea with kidney-yang deficiency syndrome, 16 characteristic genera were screened and Spearman correlation analyses were performed with CutC activity. Additionally, Spearman correlation analysis was performed to explore the associations between serum and liver TMAO levels, TNF-α, IL-6, and CutC activity. As there were no significant changes in CutC activity in small intestine contents and mucosa, the analysis focused on cecal contents and mucosal CutC activity. The correlation heatmap (Figures 9A,B) indicates that cecal contents and mucosal CutC activity, TNF-α, IL-6, serum, and liver TMAO levels are positively correlated. Specifically, cecal contents and mucosal CutC activity and IL-6 showed a significant positive correlation with serum TMAO levels, while cecal mucosal CutC activity and IL-6 exhibited a significant positive correlation with liver TMAO levels. Notably, the cecal contents CutC activity was significantly negatively correlated with *Ligilactobacillus*, while the cecal content and mucosal CutC activity showed significant positive correlations with *Negativibacillus* and *Paludicola*.

## 4 Discussion

### 4.1 Increased activity of cecal CutC induced by diarrhea with kidney-yang deficiency syndrome

The intestinal microbiota utilizes the TMA lyase complex, consisting of CutC/D, CntA/B, and YeaW/X, to metabolize choline, L-carnitine, and betaine into trimethylamine (TMA). Among these enzymes, CutC is acknowledged for its anaerobic degradation of choline, considered a primary source of TMA formation in the intestinal tract (Wang et al., 2015; Gabr and Świderek, 2020; Cai et al., 2022). CutC, a member of the glycine reductase enzyme (GRE) family, activates the glycine radical in its structure, which can generate a sulfur radical from cysteine. This sulfur radical further captures a hydrogen atom from choline’s C1, initiating a molecular rearrangement that results in TMA formation (Craciun et al., 2014; Zhu et al., 2014). TMAO is the oxidative product of TMA catalyzed by FMOs in the liver. Therefore, the role of CutC is crucial in the formation of both TMA and TMAO. In this study, a mouse diarrhea model was established by intragastric administration of adenine combined with *Folium sennae*, and the activity of CutC in the contents and mucosa of the small intestine and cecum was measured. The results revealed that there was no significant difference in CutC activity between the small intestinal contents and mucosa of the CN group, and the CD group showed similar findings. Moreover, CutC activity in the small intestinal contents and mucosa of the CD group mice was comparable to that of the CN group mice. The cecal mucosal CutC activity was consistently higher than that in the cecal content. Additionally, in comparison with the CN group, the cecal contents and mucosal CutC activity in the CD group were significantly increased. Diarrhea with kidney-yang deficiency syndrome increased the activity of CutC in the cecum while having no impact on the small intestine segment. The gut is a diverse ecosystem with distinct layered environments in different segments, leading to variations in microbial composition (de Vos et al., 2022). Moreover, TMA production is primarily localized in the cecum (Li et al., 2022d). Bacteria primarily responsible for producing CutC are mainly located in the cecum. The diarrhea with kidney-yang deficiency syndrome appears to result in an increased proliferation of bacteria producing CutC in the cecum or a decrease in the growth of bacteria that inhibit CutC. Further analysis is needed, incorporating changes in the microbial composition of cecal contents in mice.

### 4.2 Decreased beneficial bacteria and microbial dysbiosis in cecal contents as potential contributors to diarrhea with kidney-yang deficiency syndrome

The CN group exhibited a higher number of unique ASVs in cecal contents compared to the CD group, indicating a shift in bacterial composition in response to diarrhea with kidney-yang deficiency syndrome. This study further investigated the impact of diarrhea with kidney-yang deficiency syndrome on the diversity, community structure, characteristic microbial groups, and functionality of the cecal microbiota in mice. In comparison to the CN group, the CD group exhibited a decreasing trend in both Chao1 and Observed_species indices, used to assess species richness, as well as in the Shannon and Simpson indices, which evaluate species diversity. These findings indicate a reduction in species richness and diversity of cecal microbial communities in mice following adenine combined with *Folium sennae* administration. Consistent results from PCoA and NMDS analyses revealed significant differences in the community structure of cecal contents between the CN and CD groups. Earlier studies by our research group found that diarrhea with kidney-yang deficiency syndrome altered the microbial structure in the small intestine contents and mucosa of mice (Zhu et al., 2022; Li et al., 2022a), indicating distinct microbial compositions in different segments of the intestine. Therefore, it can be inferred that diarrhea with kidney-yang deficiency syndrome alters the diversity and structure of the cecal microbiota in mice.

To gain further insights into the impact of adenine combined with *Folium sennae* administration on the cecal microbiota, inter-group relative abundance comparisons were conducted. The F/B ratio is often utilized to gauge intestinal homeostasis, with an increased F/B ratio indicating an imbalance or instability in the microbial community structure (Shin et al., 2015; Li et al., 2022e). This study indicates that the F/B ratio in the CD group was higher than that in the CN group, suggesting an imbalance in the intestinal microbial environment induced by diarrhea with kidney-yang deficiency syndrome. In comparison to the CN group, the CD group exhibited a decreasing trend in the relative abundance of the *Ligilactobacillus*, and a significant reduction in the relative abundance of *Alistipes*, *Enterorhabdus*, *Desulfovibrio*, and *Candidatus_Saccharimonas*, and a significant increase in *Bacteroides* and *[Ruminococcus]_torques_group*. *Ligilactobacillus* is a lactic acid-producing probiotic in the gut, known for its various health benefits and alleviation of diarrhea (An et al., 2011; Guerrero Sanchez et al., 2022). *Alistipes*, belonging to the Bacteroidetes phylum, may have protective effects against certain diseases such as liver fibrosis, colitis, and cardiovascular diseases, and it is a bacterium that produces short-chain fatty acids (Parker et al., 2020). *Enterorhabdus* has been associated with the development of type 2 diabetes mellitus (T2DM), obesity, and non-alcoholic fatty liver disease in humans or mice (Le Roy et al., 2013; Yang et al., 2015). However, some studies have found a negative correlation between *Enterorhabdus* and pro-inflammatory cytokine concentrations, and a decrease in *Enterorhabdus* has been observed in ulcerative colitis (UC) mice (Cheng et al., 2023). *Desulfovibrio* is considered a harmful bacterium that produces hydrogen sulfide in the intestines, which is toxic to the intestinal epithelium and can cause gastrointestinal diseases (Verstreken et al., 2012). The decrease in the relative abundance of *Desulfovibrio* in diarrheal mice may be due to the inhibition of its growth by adenine or *Folium sennae*. *Candidatus_Saccharimonas*, as a probiotic, possesses anti-inflammatory properties and contributes to the secretion of IL-4 and IL-10 (Sang et al., 2020; Ge et al., 2021). *Bacteroides* is a crucial cornerstone genus in the gut microbiota and plays a significant role in the balance of intestinal microbial communities in inflammatory bowel disease, colorectal cancer, and other intestinal disorders (Garrett, 2019; Ryan et al., 2020). The elevation of *[Ruminococcus]_torques_group* has been observed in patients with diabetic retinopathy, and it is associated with sustained systemic inflammation in the irritable bowel syndrome with diarrhea (IBS-D) model (Zhen et al., 2021; Bai et al., 2022). Based on these results, it can be inferred that adenine combined with *Folium sennae* administration in mice leads to a reduction in beneficial bacteria and microbial dysbiosis in cecal contents, which may be important factors contributing to diarrhea.

### 4.3 Reduced *ligilactobacillus* in the cecal contents of mice with diarrhea with kidney-yang deficiency syndrome may elevate CutC activity, potentially leading to increased TMAO levels and promotion of inflammatory factor expression

Based on LEfSe analysis and random forest diagnostics, *Paludicola, Blautia, Negativibacillus, Paraprevotella, Harryflintia, Candidatus_Soleaferrea, Anaerotruncus, Oscillibacter, Colidextribacter, [Ruminococcus]_torques_group*, and *Bacteroides* were identified as characteristic bacteria in the CD group, while *Lachnospiraceae_UCG-006, Alistipes, Lachnospiraceae_NK4A136_group, Staphylococcus*, and *Ligilactobacillus* were characteristic in the CN group. Correlation analysis between cecal CutC activity and these characteristic bacteria revealed a significant negative correlation with *Ligilactobacillus* and a significant positive correlation with *Negativibacillus* and *Paludicola*. *Negativibacillus*, considered detrimental to health, has been found to significantly increase in patients with ulcerative colitis (Gryaznova et al., 2021), and *Paludicola*, a new genus in the Ruminococcaceae family, remains unexplored in the gut (Li et al., 2017). While *Negativibacillus and Paludicola* exhibit a positive correlation with CutC activity, further research is needed to establish the nature of their relationship. *Ligilactobacillus*, a probiotic producing lactic acid in the gut, is associated with various health benefits and diarrhea alleviation. Studies have shown that bacterial-produced lactic acid can significantly reduce TMA levels (An et al., 2011; Park et al., 2020; Guerrero Sanchez et al., 2022), and CutC activity directly influences the production of TMA, a precursor to TMAO. It can be inferred that *Ligilactobacillus* may inhibit CutC activity and reduce TMA production by generating lactic acid. The negative correlation between *Ligilactobacillus* and cecal CutC activity suggests that the high CutC activity in cecal contents induced by diarrhea with kidney-yang deficiency syndrome is mediated by the reduction of *Ligilactobacillus*.

IL-6 and TNF-α are inflammatory factors critical in both acute and chronic inflammation (Gabay, 2006). TMAO, known for its pro-inflammatory properties, is implicated in various diseases, particularly atherosclerosis, chronic kidney disease, and type 2 diabetes (Tang et al., 2015; Janeiro et al., 2018; Zhuang et al., 2019; Tan et al., 2020). Correlation analysis was conducted between TMAO levels, inflammatory markers, and cecal CutC activity. The results revealed a positive correlation between TMAO levels and IL-6, TNF-α, and cecal CutC activity, with a significant positive correlation observed between TMAO levels and IL-6, as well as between TMAO levels and cecal CutC activity. Previous research has demonstrated that increased TMAO induces the production of IL-6 and TNF-α, exacerbating peritoneal inflammation in peritoneal dialysis patients and increasing the risk of peritonitis. This mechanism may involve TMAO inducing macrophages toward M1 polarization, leading to increased expression of IL-6 and TNF-α (Wu et al., 2020; Zhang et al., 2022), aligning with the findings of this study.

## 5 Conclusion

In summary, diarrhea with kidney-yang deficiency syndrome significantly affects the physiological status, digestive enzyme activity, CutC activity, TMAO levels, and inflammatory response in mice. Additionally, there are changes in the composition and function of cecal microbiota, indicating an important impact of diarrhea with kidney-yang deficiency syndrome on the host intestinal microbiota balance. The occurrence of diarrhea with kidney-yang deficiency syndrome may be associated with dysbiosis of intestinal microbiota, increased CutC activity, elevated TMAO levels, and heightened inflammatory factor levels. These findings not only contribute to a deeper understanding of the pathogenesis of diarrhea but also provide important theoretical and experimental foundations for further research and treatment of diarrhea with kidney-yang deficiency syndrome.

## Data availability statement

The datasets presented in this study can be found in online repositories. The names of the repository/repositories and accession number(s) can be found at: https://www.ncbi.nlm.nih.gov/, PRJNA1041605.

## Ethics statement

The animal study was approved by the Animal Ethics and Welfare Committee of Hunan University of Chinese Medicine. The study was conducted in accordance with the local legislation and institutional requirements.

## Author contributions

MG: Conceptualization, Data curation, Writing – original draft. LF: Data curation, Methodology, Writing – review & editing. MC: Investigation, Visualization, Writing – review & editing. JS: Investigation, Visualization, Writing – review & editing. ZT: Funding acquisition, Supervision, Writing – review & editing. WH: Funding acquisition, Supervision, Writing – review & editing.

## Footnotes

^1^https://www.ncbi.nlm.nih.gov/bioproject/PRJNA1041605
